# Nomenclature and treatment of secondary urethral strictures following primary hypospadias repair: weighing up academic principles and clinical pragmatism

**DOI:** 10.1007/s00345-020-03472-w

**Published:** 2020-10-06

**Authors:** Malte W. Vetterlein, Valentin Zumstein, Luis A. Kluth, Silke Riechardt, Roland Dahlem, Margit Fisch

**Affiliations:** 1 Department of Urology, University Medical Center Hamburg-Eppendorf, Hamburg, Germany; 2 Department of Urology, Cantonal Medical Center St. Gallen, St. Gallen, Switzerland; 3 Department of Urology, University Medical Center Frankfurt, Frankfurt/Main, Germany

Dear Editor,

With great interest we have read the comments by Drs. Shekar and Shivakumar [1] regarding our recent article on buccal mucosal graft urethroplasty (BMGU) for distal urethral strictures [2]. In that regard, we would like to put some points in perspective:

First, the authors have expressed some criticism about including patients who had previously undergone treatment for hypospadias. Hence, they claim that our report of a “homogeneous” cohort was untrue. While we concur with the notion that patients with hypospadias-associated strictures do embody a very specific subgroup with distinct disease characteristics, we would like to underline that the homogeneity of our series is warranted by the surgical technique (BMGU using a dorsal inlay) [2]. Contrary to the authors’ opinion, we believe that the end does actually justify the means and that it is important to emphasize the feasibility and durability of one particular surgical technique in such population, especially given the abundance of techniques for the repair of the distal urethra. From a clinical perspective, we are confident that it is reasonable to pragmatically approach and consider all patients for the decision-making process who present with urethral narrowing of the fossa navicularis or meatus, irrespective of the etiology. In our opinion, the nomenclature is of secondary importance here. Whether the urethral condition is labeled “obliteration”, “narrowing”, “stricture” or “stenosis” does not change the fact that the patient is in need of surgical intervention. Thus, the controversy regarding the correct nomenclature is more or less predominantly academic [3]. However, we absolutely agree that using internationally acknowledged staging systems might facilitate multi-institutional comparisons, first and foremost to evaluate the efficacy of different surgical techniques in discrete stricture cohorts. A great example is the recently developed LSE classification system based on stricture length (L), segment (S), and etiology (E) [4], which particularly accounts for a stricture in the segment of prior hypospadias repair and allows for organizing “[…] a heterogeneous condition […] that will improve our ability to study the disease process” [4].

**Fig. 1:**
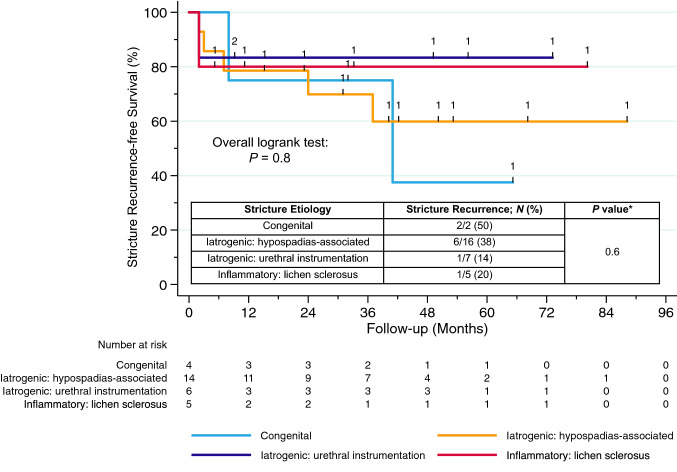
Kaplan–Meier estimates of stricture recurrence-free survival in 32 men who underwent single-stage dorsal inlay BMGU stratified by stricture etiology (*N* = 3 were omitted due to short follow-up of < 2 months). *BMGU* buccal mucosal graft urethroplasty. *Fisher’s exact test.

Second, as the editorialists correctly mention, we had initially refrained from stratifying stricture recurrence by etiology given the relatively small sample of 32 patients with only 10 recurrences and thus, limited room for interpretation. Nevertheless, we gladly provide those data here to complete the full picture and the results are depicted in Fig. 1. Overall, there was no statistical difference in recurrence rates between different etiologies (*P ≥ *0.6). However, it should be noted that it is impossible to draw statistically meaningful conclusions from stratifying those ten patients according to etiology and larger study cohorts are needed to allow for valid inferences.

Finally, we entirely agree with the authors that eventually long-term outcomes should be reported to evaluate the durability of a surgical technique. Indeed, we have recently presented preliminary results from a cohort of 81 patients after primary hypospadias repair that underwent one-stage BMGU for secondary stricture. At a median follow-up of 70 months, 2-year and 5-year recurrence-free survival was 70% and 56%, respectively, which translated into 5-year recurrence-free survival of 65% in the subgroup of patients presenting with meatal stenosis or fossa navicularis stricture [5]. Such data along with endeavors to collapse multi-institutional databases will help to better understand the distinct features and therapeutic requirements of patients with rare stricture locations, adverse etiology, and other complicating factors by increasing sample sizes and duration of follow-up.
